# Donor derived cell free DNA in lung transplant recipients rises in setting of allograft instability

**DOI:** 10.3389/frtra.2024.1497374

**Published:** 2024-12-16

**Authors:** Joshua B. Smith, Ryan A. Peterson, Raymond Pomponio, Mark Steele, Alice L. Gray

**Affiliations:** ^1^ Division of Pulmonary Sciences and Critical Care, University of Colorado School of Medicine, Aurora, CO, United States; ^2^ Department of Biostatistics and Informatics, Center for Innovative Design and Analysis, University of Colorado School of Public Health, Aurora, CO, United States

**Keywords:** lung allograft monitoring, donor derived cell free DNA, lung transplant biomarker, thoracic transplantation, non-invasive monitoring

## Abstract

**Purpose:**

The purpose of this study was to evaluate the correlation between longitudinal monitoring of donor-derived cell free DNA (dd-cfDNA) in lung transplant recipients and a “gold standard” of existing tools (pulmonary function testing, radiographic imaging, laboratory and bronchoscopy data, clinical judgment) to assess allograft function.

**Methods:**

24 consecutive transplant recipients were prospectively enrolled in this study measuring dd-cfDNA levels monthly in the first year after bilateral lung transplant. Blinded clinical adjudications were performed at the same timepoints to categorize allograft function as stable (FEV1 within 10% of prior value or when compared to best two averaged post-transplant values) or unstable. When deemed unstable, etiology of unstable graft function was elicited based on available clinical data. We then evaluated the association between dd-cfDNA and the clinical impression of allograft function using linear mixed models which adjusted for patient-level covariates and time since transplant.

**Results:**

Unstable allografts were associated with 54.4% higher measures of dd-cfDNA, controlling for time since transplant and demographic covariates [*adjusted mean ratio (aMR)* = 1.54, 95% CI: 1.25–1.91]. Females tended to have higher measures of dd-cfDNA (*aMR* = 1.90 95%CI: 1.14–3.16). A two-fold increase in dd-cfDNA was associated with declines in FEV1 and FVC of 0.047 and 0.066 L, respectively, controlling for time since transplant and demographic covariates (*slope:* −0.047 95%CI: −0.076 to −0.019, and *slope:* −0.066 95%CI: −0.097 to −0.035, respectively). Discussion: Donor derived cell free DNA presents a potential additional minimally invasive clinical tool in lung transplant allograft monitoring within the first year of transplant, with unstable allografts correlating with higher dd-cfDNA values.

## 1 Introduction

Lung transplantation is a viable treatment option for many individuals with end stage lung diseases. While short term outcomes have improved with evolving surgical and perioperative techniques, long term outcomes remain essentially unchanged, due in large part to the development of chronic lung allograft dysfunction (CLAD) (1). With no proven effective treatments for CLAD, transplant pulmonologists monitor lung transplant recipients closely to assess allograft function to try to intervene earlier in the disease course when the process is ostensibly more inflammatory and potentially responsive to intervention in an effort to alter the trajectory of the disease.

The current “gold standard” for assessing lung allograft function is the treating transplant pulmonologist's clinical impression, an amalgam of data including, pulmonary function testing, radiographic imaging, bronchoscopy with transbronchial biopsies and lab results. More sophisticated ways of monitoring the lung allograft are needed to identify those at highest risk for developing CLAD and those who are in a more immunologically quiescent state after their lung transplant.

Prior studies looking at cfDNA monitoring in lung transplant have shown the test correlates with lung transplant rejection, but its use as a diagnostic test is less clear (2). Keller et al. used results of donor-derived cell free DNA (dd-cfDNA) testing to guide decision-making regarding need for invasive testing (such as bronchoscopy) and found increased rates of acute lung allograft dysfunction (ALAD) in those with elevations in dd-cfDNA (3). We wanted to see if and how the dd-cfDNA test correlated with the physician's assessment of the allograft, and if this correlation was also associated with lung function and CLAD at 1 year.

## 2 Materials and methods

**Figure 1:**
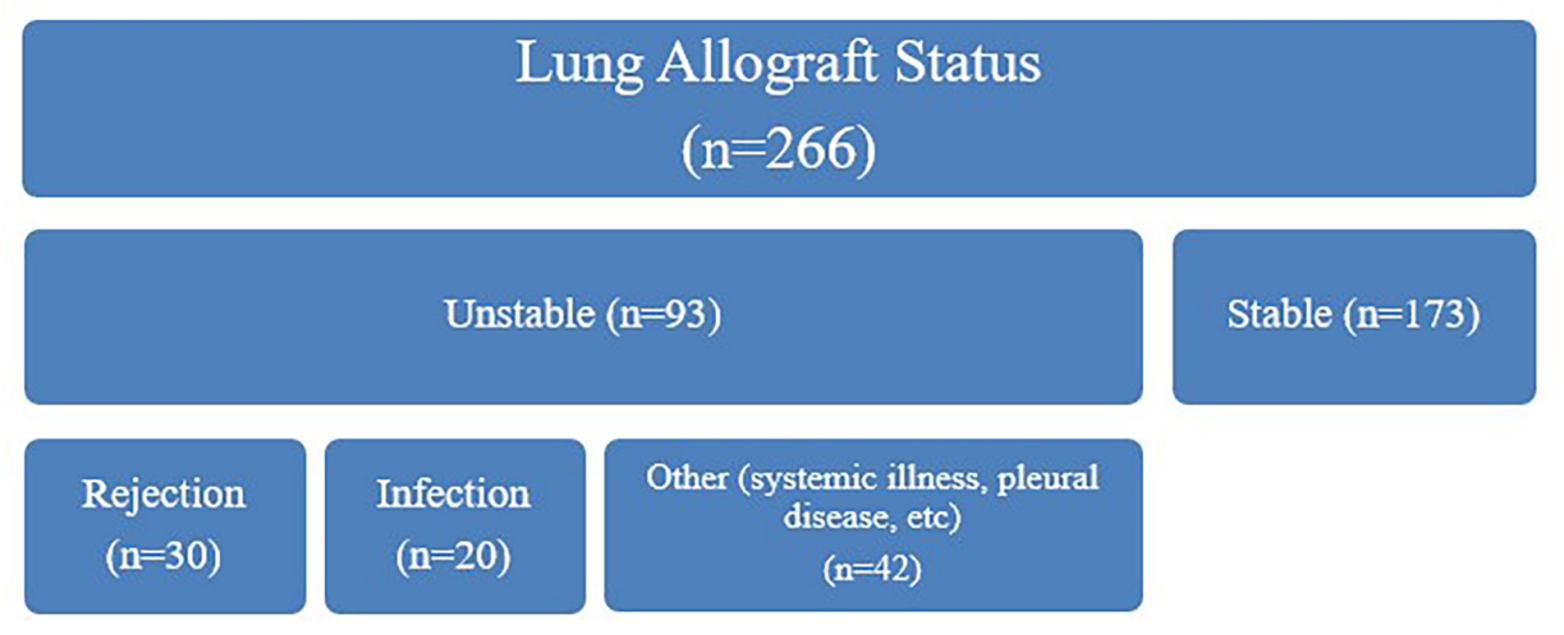
Clinical adjudication of lung allografts. Allograft status was first adjudicated at each timepoint by two clinicians to either stable (FEV1 within 10% of prior value or averaged two post-transplant highest values) or unstable (based on either a decline in FEV1 of ≥10% from most recent value or averaged best post-transplant values, or if this qualifier was not met, then another compelling clinical indication of graft instability such as a positive transbronchial biopsy or imaging abnormality). If deemed unstable the underlying etiology was further determined based on available clinical information, including history, infectious studies, imaging, and bronchoscopy if available. Note that one unstable allograft was not adjudicated to rejection, infection, or “other”.

Twenty-four adult bilateral lung transplant recipients were enrolled in the study at the time of transplant between February 28, 2020 and November 7, 2020. Anyone with history of prior organ transplant was excluded. Blood samples were obtained monthly. Patients also underwent programmatic standard of care clinical assessments which included a monthly visit with the transplant pulmonologist, spirometry, chest imaging, and routine blood work for the first 12 months after transplant (Figure 1). Bronchoscopy with transbronchial lung biopsy was performed at post-operative 1, 3, 6, 9, and 12 month visits. Additionally, clinically indicated bronchoscopies were performed based on a decline in spirometry, concerning radiographic features, or respiratory symptoms. Blood samples were sent for assessment of dd-cfDNA. At each time point, two of the three clinical investigators were asked to assess impression of allograft function once all clinical information had resulted, based on Figure 1, without access to or knowledge of the results of the dd-cfDNA levels. The clinician adjudicators were asked to determine any contributing conditions, and as such patients may have had multiple contributing factors identified in their assessment. If both investigators were concordant in their assessment, that impression was deemed the final adjudication. If there was discordance, the three clinical investigators reviewed the patient's information together to reach a consensus adjudication.

Longitudinal measures were collected monthly for up to one-year post-transplant; measures included semi-quantitative dd-cfDNA measured by Allosure (CareDx), forced expiratory volume (FEV 1 s), forced vital capacity (FVC), and adjudicated lung allograft status. This study was approved by the Colorado Multiple Institutional Review Board (COMIRB 19-2946).

### 2.1 Statistical analysis

We first assessed the marginal difference in dd-cfDNA measures between stable vs. unstable grafts using a linear mixed model with subject-specific random intercepts to account for repeated measures within a subject.

We used a linear mixed model for our primary outcome of log-transformed dd-cfDNA with fixed effects for time since transplant, lung allograft status, and demographic covariates (age, sex, native disease) and subject-specific random intercepts and slopes. We report estimated coefficients on the exponentiated scale to aid interpretability (exponentiated coefficients can be interpreted as adjusted mean ratios, aMR). We report model estimates for the variance of the random effects, as well as intra-class correlation coefficient (ICC).

We modeled FEV1 and FVC as secondary outcomes to evaluate the association between pulmonary function testing and dd-cfDNA in a similar mixed modeling framework. The predictors were identical to the primary model except that we did not include lung allograft status as a predictor, since it is definitionally related to pulmonary function. We transformed dd-cfDNA with base-2 logarithms (rather than natural logarithms) to aid interpretability. Both models included subject-specific random intercepts and slopes.

For generating and visualizing predicted probabilities of allograft stability, we used a binomial-family logit-link generalized linear mixed model with adjudicated lung allograft status as the binary outcome, fixed effects for time since transplant, log-transformed dd-cfDNA, baseline age, sex, and listing diagnosis, and random intercepts to account for repeated measures within a subject.

Statistical modeling was computed in the R programming language version 4.2.1 (4) with the lme4 package (5). Models were fit using restricted maximum likelihood estimation. In the primary outcome model, 51 of 276 monthly timepoints (18.4%) were missing allograft status or dd-cfDNA. In the secondary outcome models, 60 of 276 timepoints (21.7%) were missing dd-cfDNA or PFT values. Missing values were excluded from presented models; a sensitivity analysis was performed using multiple imputation by chained equations with 25 imputed data sets via the mice R package (6).

## 3 Results

**Figure 2:**
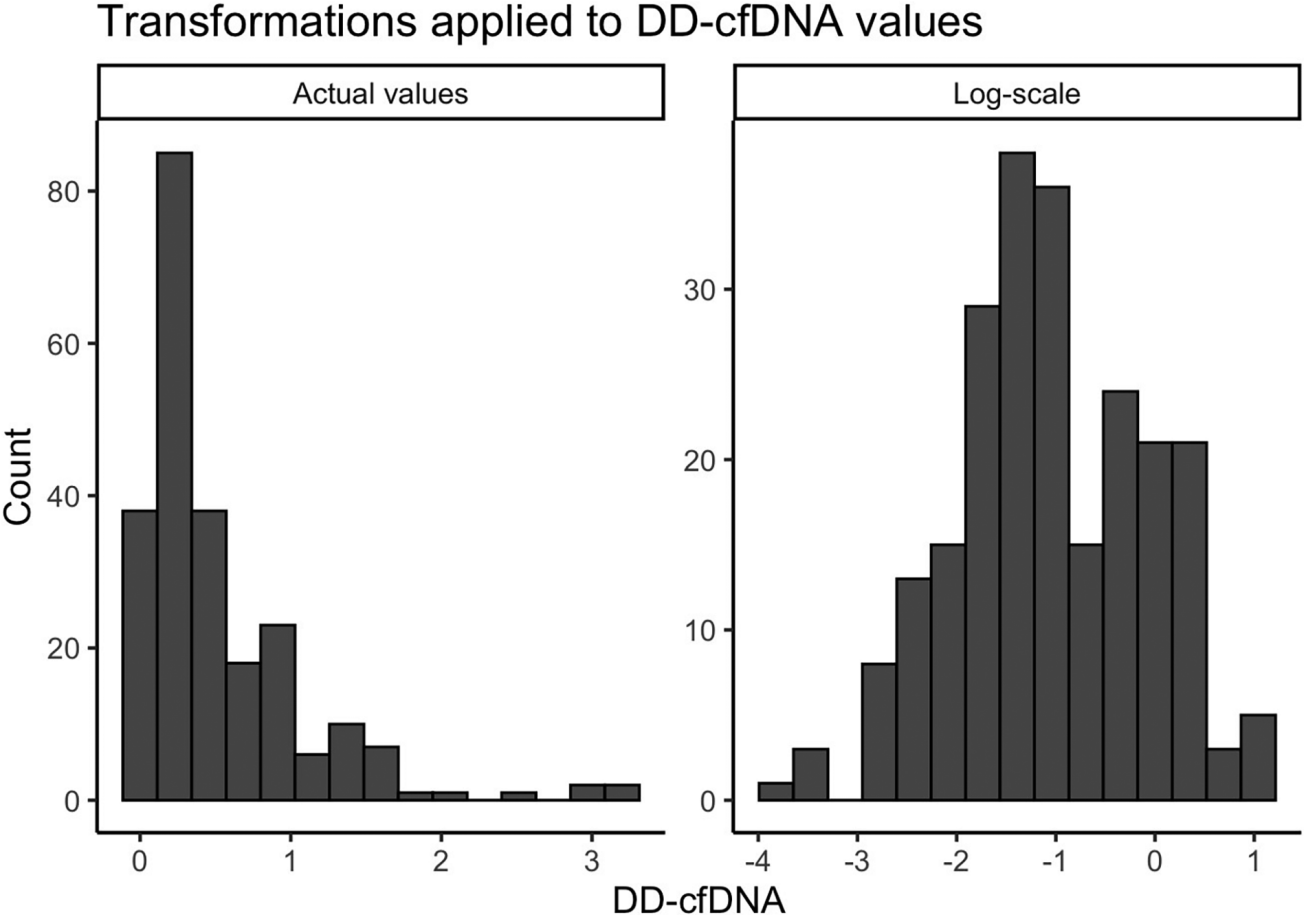
Transformations applied to dd-cfDNA values. A small constant (0.025) was added to the original dd-cfDNA values due to the presence of zeros.

Descriptive demographic statistics for the study cohort are displayed in Table 1. The study cohort's demographics (sex, race, ethnicity, baseline age, and listing diagnosis) are described using summary statistics (means/standard deviations for continuous variables, counts/percentages for categorical variables). The distribution of donor-derived cell-free DNA measures were found to be heavily right-skewed, and we determined that a logarithmic transformation helped to normalize these values prior to modeling (Figure 2). We also produced visualizations and summary statistics of dd-cfDNA stratified by sex to help describe our observed sex-specific differences.

**Table 1:** Summary of demographics.

Characteristic	*N* = 24
Sex	
Female	13 (54%)
Male	11 (46%)
Race	
Black/African American	1 (4.2%)
Hispanic/Latino	1 (4.2%)
Other	1 (4.2%)
White/Caucasian	21 (88%)
Ethnicity	
Hispanic	4 (17%)
Non Hispanic	20 (83%)
Age at time of transplant	60.5 (59.0, 62.0)
Listing diagnosis	
COPD	15 (62%)
Interstitial lung disease	9 (38%)
Deceased	1 (4.2%)
Number of unstable allografts	3 (2, 5)

Note: Median (IQR) reported for age, number of unstable grafts. One subject was deceased at Month 9.

Mortality was expected to be 12% (7), or 3 subjects, but only one subject died during the study. The majority of subjects in this cohort had an underlying disease of COPD (62%), and all other patients had interstitial lung disease; no patients had cystic fibrosis or primary pulmonary hypertension, making this cohort slightly different from national trends.

**Figure 3:**
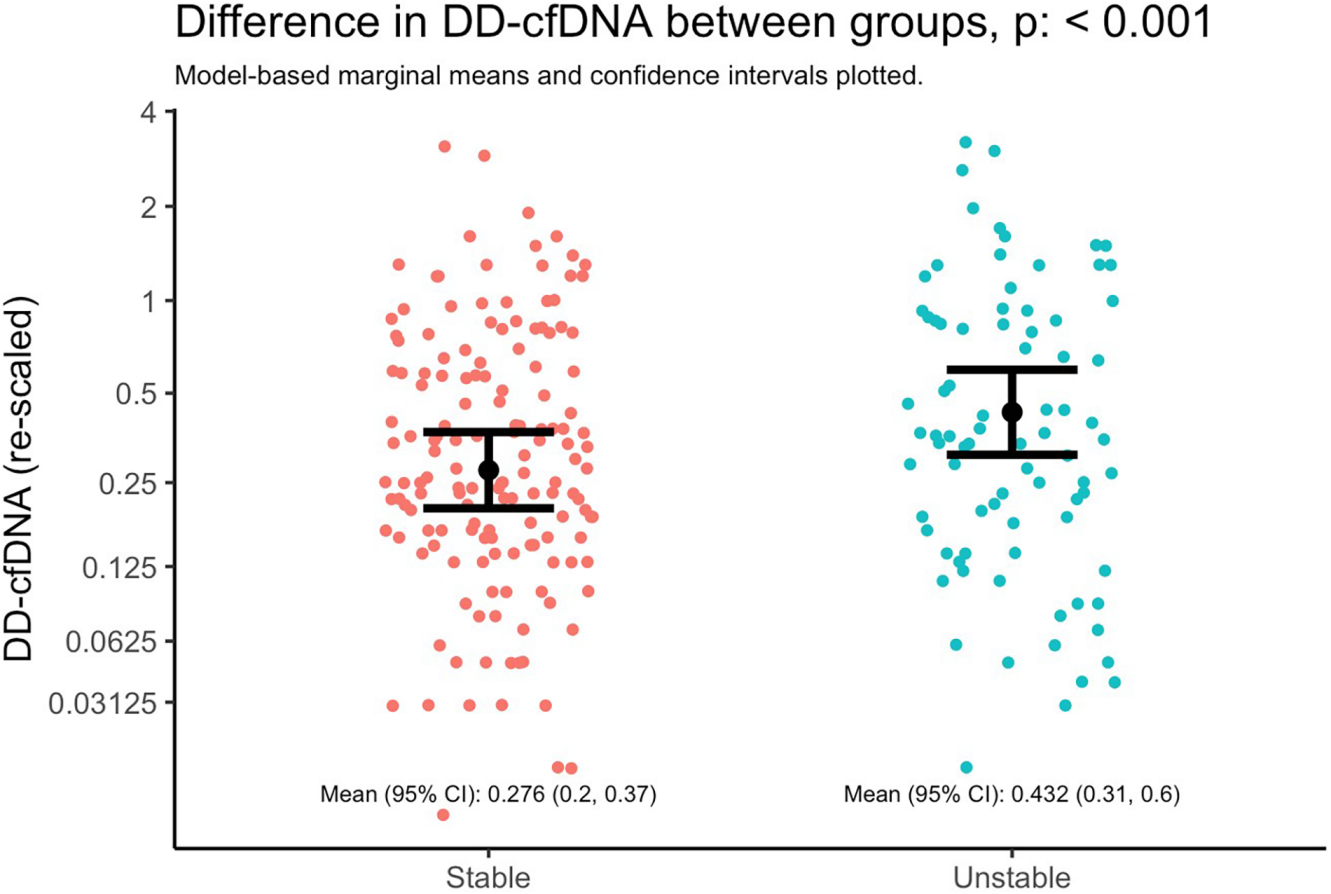
Difference in dd-cfDNA between stable and unstable allografts. Marginal means and 95% CIs from a linear mixed model with dd-cfDNA values as the outcome and lung stability status as the primary and only explanatory variable. The model accounts for correlated measures within individual subjects via subject-specific random intercepts. Note that the y-axis is plotted with the original units of dd-cfDNA values (i.e., not log-transformed units), but it is re-scaled by orders of magnitude to illustrate differences as if on the base-2 logarithm scale; the distance between y-axis tick marks can be interpreted as doubling the value of dd-cfDNA.

**Figure 4:**
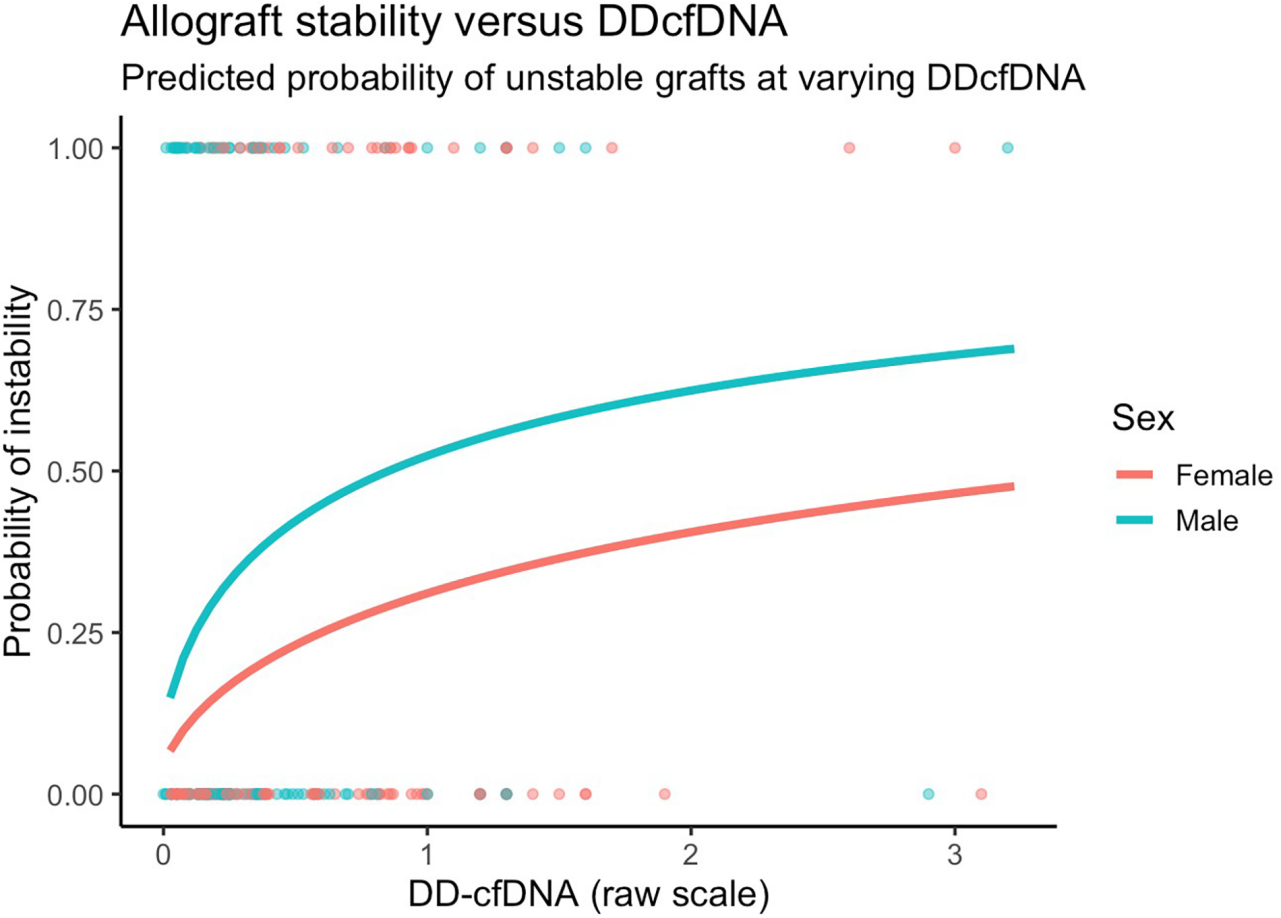
Model predicted probabilities of unstable grafts by sex and dd-cfDNA values. Predictions presented are for 60-year old patients with COPD six months after transplant.

A total of 266 dd-cfDNA samples were obtained and adjudicated based on allograft status. Of these cases, 173 were assessed to have stable allograft function and 93 were assessed to have unstable allograft function. Among unstable allografts, 30 were clinically adjudicated to be due to rejection, 20 due to infection, 42 were categorized as “other”, and 1 was missing this adjudication. The median number of clinically adjudicated unstable allografts per subject were 3 (IQR 2,5). We observed a significant difference in dd-cfDNA levels based on sex, with higher levels observed in females (Figures 3, 4). The median dd-cfDNA in female subjects was 0.38 (IQR 0.20, 0.86) and in male subjects was 0.25 (IQR 0.13, 0.47).

Donor derived cell-free DNA levels was higher in the setting of an unstable allograft (Figure 3), adjusting for time since transplant and subject-specific intercepts. Specifically, the mean dd-cfDNA at stable timepoints was 0.276 (95% CI 0.2, 0.37) and at unstable timepoints was 0.432 (95% CI 0.31, 0.6); mean ratio: 1.52 (95% CI: 1.19, 1.94; *p* = 0.00085).

After adjusting for covariates, we found similarly that changes in dd-cfDNA correlate with allograft instability**.** Specifically, a doubling in dd-cfDNA was associated with 52.4% higher adjusted odds of an unstable allograft (95% CI: 12.7% to 106.1%, *p* = 0.006). Allograft instability was not associated with sex after controlling for other covariates (Figure 4).

For the primary outcome of dd-cfDNA, and controlling for time since transplant, gender, and subject-specific random effects, an unstable allograft was associated with a 1.54 times greater dd-cfDNA compared to a stable allograft, that is a 54.4% increase (95% CI: 24.7%–91.3%, *p* < 0.001). Females tended to have 89.9% higher dd-cfDNA when compared to males (95% CI: 13.9%–216.2%, *p* = 0.014, Table 2).

**Table 2:** Mixed model predictors of dd-cfDNA.

Predictors	Log dd-cf DNA		
	aMR	CI	*p*
Months after transplant	0.934	0.889–0.981	**0.007**
Unstable graft status	1.544	1.247–1.913	**<0.001**
Age at baseline	1.020	0.970–1.072	0.442
Gender: Female	1.898	1.139–3.162	**0.014**
Diagnosis: COPD	1.046	0.635–1.724	0.859
Random Effects	Estimate		
*σ*^2^ (Residual variance)	0.43		
Random intercept variance	0.94		
Random slope variance	0.01		
Correlation between random int. and slope	−0.83		
Intra-class correlation	0.51		
*N*_subjects_	23		
Observations	225		
Marginal R^2^/Conditional R^2^	0.166/0.592		

Note: Exponentiated Coefficients. Results from a linear mixed model with the primary outcome of (log-transformed) dd-cfDNA and predictors of interest (fixed effects). Random effects include subject-specific slopes and intercepts. aMR: adjusted mean ratio; CI: 95% confidence interval.

Bolded values are statistically significant, with a *p*-value of <0.05.

A 10-fold increase in dd-cfDNA was associated with a 158 ml decrease in FEV1 (95% CI: 63, 252; *p* = 0.001) and a 218 ml decrease in FVC (95% CI: 115, 321; *p* < 0.001), adjusting for age,sex, diagnosis, months after treatment, and subject-specific intercepts and slopes. An increase such as this was associated with a 4.1 factor increase in the odds of graft instability (95% CI: 1.5, 11.1; *p* = 0.006) adjusted for age, sex, diagnosis, months after transplant, and subject-specific intercepts. On a month-to-month basis this was not a frequent occurrence (1.98% of the time), however the course of the 12 month study period, it occurred in 47.8% of patients. This suggests that single time points are not necessarily the most important values, but rather changes over time may indicate a graft at risk when spirometric changes are not dramatic.

In secondary models for lung function, we found that dd-cfDNA correlated with lung function as represented by both FEV1 and FVC (Supplementary Figure 1). Controlling for time since transplant, gender, and subject-specific random effects, a doubling in dd-cfDNA was associated with a decline in FEV1 of 0.047 L (95% CI: −0.076 to −0.019, *p* = 0.001) (Table 3). Females tended to have lower FEV1 by 0.657 L (*p* = 0.027) and FVC by 0.852 L (*p* = 0.011), on average, likely due to differences in height. Controlling for time since transplant, gender, and subject-specific random effects, a doubling in dd-cfDNA was associated with a decline in FVC of 0.066 L (95% CI: −0.097 to −0.035, *p* < 0.001). All reported findings were qualitatively similar in multiple imputation sensitivity analyses.

**Table 3:** DD-cfDNA as a predictor of lung function

Predictors	FEV 1			FVC		
	Slope	CI	*p*	Slope	CI	*p*
Base-2 Log DD-cf DNA	−0.047	−0.076 to −0.019	**0.001**	−0.066	−0.097 to −0.035	**<0.001**
Months after transplant	0.025	0.008–0.043	**0.005**	0.044	0.021–0.066	**<0.001**
Age at baseline	0.005	−0.052–0.062	0.870	0.011	−0.053–0.075	0.739
Gender: Female	−0.657	−1.239 to −0.075	**0.027**	−0.852	−1.505 to −0.199	**0.011**
Diagnosis: COPD	0.312	−0.258–0.882	0.282	0.136	−0.503–0.775	0.676
Random Effects						
σ^2^ (Residual variance)	0.04			0.04		
Random intercept variance	0.43			0.55		
Random slope variance	< 0.01			< 0.01		
Correlation between random int. and slope	0.15			0.09		
Intra-class correlation	0.94			0.94		
N _subjects_	23			23		
Observations	216			216		
Marginal R^2^/Conditional R^2^	0.226/0.950			0.261/0.957		

Statistically significant values are indicated in bold.

## 4 Discussion

We investigated the strength of the association between concurrent dd-cfDNA levels and allograft status, controlling for clinically relevant covariates as well as within-subject correlation (i.e., mixed modeling). In this cohort, those with unstable allograft statuses had higher dd-cfDNA levels. Using models to account for repeated measures, we found that unstable allografts were associated with 54.4% higher measures of dd-cfDNA, controlling for time since transplant and demographic covariates (*Slope* = 1.544, 95%CI: 1.247–1.913).

Using similar methods, evidence of associations were found between dd-cfDNA values and FEV1 and FVC outcomes, measured in liters. A doubling in dd-cfDNA was associated with declines in FEV1 and FVC of 0.047 and 0.066 liters, respectively (controlling for time since transplant and demographic covariates). A decline such as this would not necessarily be identified as clinically significant, and therefore points to a potential benefit of incorporating dd-cfDNA monitoring into routine practice. In the face of apparently stable lung function or subtle declines of unclear significance, an increase in dd-cfDNA might prompt further investigation and provide a window of opportunity for therapeutic interventions to preserve allograft function. Similarly, a doubling in dd-cfDNA values was associated with 52.4% higher odds of allograft instability (controlling for time since transplant and demographic covariates). This corroborates earlier studies demonstrating a correlation of elevation in dd-cfDNA with insults to the allograft (2, 8).

Determining a clinically significant threshold is challenging, as the 10% decline identified in current working definitions of acute lung allograft dysfunction represent different values depending on the patient's baseline. We found that a 10-fold increase in dd-cfDNA was associated with 158 ml decline in FEV1 and 218 ml decline in FVC; under some circumstances this may meet the 10% threshold for ALAD but in other patients it may still be conventionally deemed subclinical. This does suggest that trending serial dd-cfDNA measurements may provide insight into the allograft status, instead of using a single data point to make an assessment.

Females in this cohort had 89.8% higher measures of dd-cfDNA (*Slope* = 1.898, 95%CI: 1.139–3.162). To our knowledge, such sex differences in dd-cfDNA levels have not previously been reported, so the significance of this finding is uncertain and may be related to small sample size.

This study shows that dd-cfDNA is a meaningful biomarker that correlates with overall lung allograft function, and not just useful in detecting acute insults such as acute rejection or infections. It may be that monitoring dd-cfDNA over time is more sensitive than spirometry in identifying those at greater risk of CLAD development. Serial surveillance of dd-cfDNA to detect increases over time may provide more diagnostic utility to the provider than using a single result. Monitoring dd-cfDNA likely has utility in characterizing relatively subtle changes in lung function, before FEV1 meets the Δ10% change would conventionally denote an unstable allograft, or one that needs further investigation. The low incidence of mortality, and CLAD, in our group limit our assessment of this, and larger, longer-term studies would be needed to better understand the implications of dd-cfDNA monitoring in association with the development of CLAD.

The advantage of serologic surveillance technology in lung transplant is that it is minimally invasive and less prone to the confounding factors associated with spirometry such as technique, effort, and pleural disease. Additionally, it can be used for remote monitoring of patients. This study investigated patients within the first year of transplant, where engagement with the transplant providers is most frequent. An important question to answer in the future is how this technology can play a role in long-term graft monitoring.

The small sample size of this cohort limits the ability to reliably associate increases in dd-cfDNA with specific graft insults, such as to what degree dd-cfDNA correlates with ACR or AMR, for example. Additional limitations include missing data, which we assume is missing at random, and the possibility for unobserved confounding factors that could cause dd-cfDNA and outcomes to change in tandem. Another question that would be better characterized by a larger study would be the ability to distinguish non-immunologic causes of graft instability from immunologic etiologies. This is an important distinction, because if dd-cfDNA demonstrates utility in distinguishing between these different causes, it could then allow for personalized interventions without necessarily requiring augmentation of immunosuppression for all-comers with graft dysfunction. The advantage of this study is that it clearly demonstrates a correlation between dd-cfDNA and the transplant pulmonologist's clinical impression of lung allograft function, when assessed in a blinded fashion, something that has not previously been shown. It shows that not only is dd-cfDNA a useful research tool, but it also has one with clear potential clinical utility. Larger, multicenter studies are needed to reveal the full potential.

## Data Availability

The raw data supporting the conclusions of this article will be made available by the authors, without undue reservation.
