# Supplementary material for: Donor derived cell free DNA in lung transplant recipients rises in setting of allograft instability
Source: Front Transplant. 2024 Dec 16;3:1497374. doi: 10.3389/frtra.2024.1497374 (PMC11683734; doi:10.3389/frtra.2024.1497374)

Supplementary Figure 1. DD-cfDNA correlation with FEV1 and FVC. Predictions presented are for 60-year old patients with COPD six months after transplant.

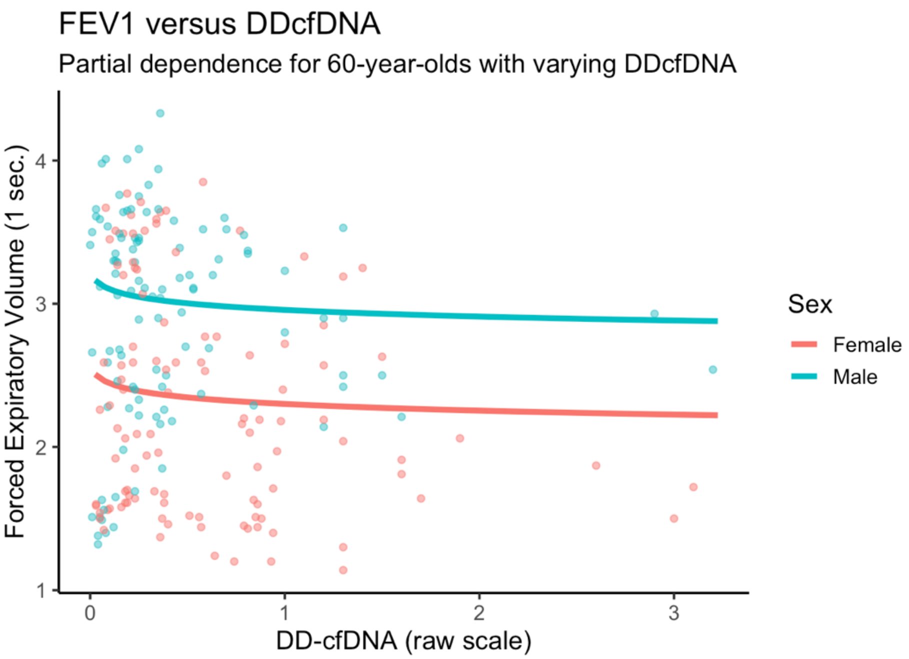

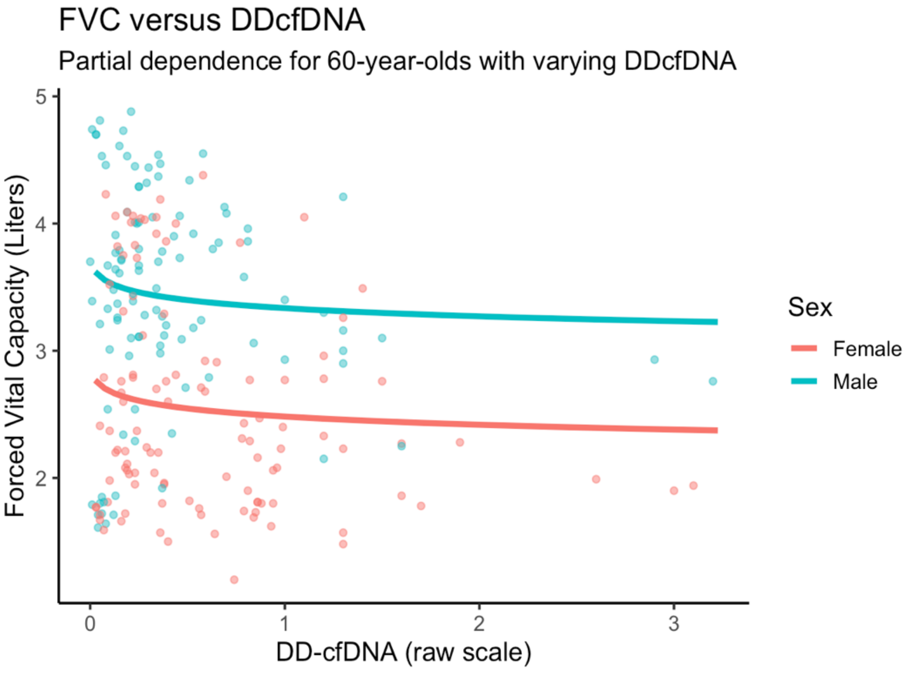

Supplement: Supplementary file 1 [file Supplementaryfigure1.pdf]
